# Infection of Feral Phenotype Swine with Japanese Encephalitis Virus

**DOI:** 10.1089/vbz.2023.0030

**Published:** 2023-11-28

**Authors:** So Lee Park, Yan-Jang S. Huang, Amy C. Lyons, Victoria B. Ayers, Susan M. Hettenbach, D. Scott McVey, Leela E. Noronha, Kenneth R. Burton, Stephen Higgs, Dana L. Vanlandingham

**Affiliations:** ^1^ Department of Diagnostic Medicine and Pathobiology, College of Veterinary Medicine, Kansas State University, Manhattan, Kansas, USA.; ^2^ Biosecurity Research Institute, Kansas State University, Manhattan, Kansas, USA.; ^3^ School of Veterinary Medicine and Biomedical Sciences, University of Nebraska-Lincoln, Lincoln, Nebraska, USA.; ^4^ Arthropod-Borne Animal Diseases Research Unit, Center for Grain and Animal Health Research, Agricultural Research Service, United States Department of Agriculture, Manhattan, Kansas, USA.

**Keywords:** feral pig, amplifying host, Japanese encephalitis virus

## Abstract

**Background:**

Japanese encephalitis virus (JEV) is a mosquito-borne zoonotic flavivirus and the leading cause of pediatric encephalitis in the Asian Pacific region. The transmission cycle primarily involves *Culex* spp. mosquitoes and Ardeid birds, with domestic pigs (*Sus scrofa domestica*) being the source of infectious viruses for the spillover of JEV from the natural endemic transmission cycle into the human population. Although many studies have concluded that domestic pigs play an important role in the transmission cycle of JEV, and infection of humans, the role of feral pigs in the transmission of JEV remains unclear. Since domestic and feral pigs are the same species, and because feral pig populations in the United States are increasing and expanding geographically, the current study aimed to test the hypothesis that if JEV were introduced into the United States, feral pigs might play a role in the transmission cycle.

**Materials and Methods:**

Sinclair miniature pigs, that exhibit the feral phenotype, were intradermally inoculated with JEV genotype Ib. These pigs were derived from crossing miniature domestic pig with four strains of feral pigs and were used since obtaining feral swine was not possible.

**Results:**

The Sinclair miniature pigs became viremic and displayed pathological outcomes similar to those observed in domestic swine.

**Conclusion:**

Based on these findings, we conclude that in the event of JEV being introduced into the United States, feral pig populations could contribute to establishment and maintenance of a transmission cycle of JEV and could lead to the virus becoming endemic in the United States.

## Introduction

Japanese encephalitis virus (JEV) is a zoonotic flavivirus primarily transmitted by *Culex tritaeniorhynchus* mosquitoes in the Asia Pacific region. Enzootic transmission of JEV involves water birds in the Ardeidae family, resembling the use of avian amplifying hosts in the maintenance of mosquito-borne encephalitic flaviviruses (Boyle et al., 1983; Buescher et al., 1959; Le Flohic et al., 2013). Epizootic outbreaks of JEV are often associated with agricultural activities in the endemic region because domestic pigs (*Sus scrofa domesticus*) can develop viremia for the transmission by competent mosquitoes. Indeed, many human Japanese encephalitis cases reported in Southeast Asia are proximal to pig farms or rice fields, which support the breeding of *C. tritaeniorhynchus* in nature.

Modern domestic pigs are a subspecies *S. scrofa domesticus* originated from the domestication of feral pigs or wild boars (*S. scrofa*). Feral pigs are found in large numbers in nature and are one of the most common ungulates in the world (Ruiz-Fons, 2017). Interbreeding between domestic pigs and feral pigs frequently takes place around the world, including in several JEV endemic countries. The high seroprevalence rates, ranging from 66% to 83%, and RNA isolation of wild boars sampled from Korea and Japan show that feral pigs in the Asia Pacific region can be exposed to JEV in nature (Nidaira et al., 2014; Nidaira et al., 2007; Nidaira et al., 2008; Ohno et al., 2009; Tan et al., 2012; Yang et al., 2012). However, it is unclear whether or not feral pigs can develop viremia to sustain the enzootic transmission of JEV (Hamano et al., 2007; Nidaira et al., 2014; Ruiz-Fons, 2017).

In contrast to various studies that have shown the uniformed susceptibility of domestic pigs to JEV, no published reports have examined the kinetics of JEV infection in either feral pigs or domestic-feral pig hybrids, presumably due to the safety and biosecurity concerns. The lack of such knowledge precludes the understanding of how feral pigs can sustain the enzootic transmission of JEV, especially in Southeast Asian countries where pig farming is uncommon but human JE is often reported, for example, Indonesia and Malaysia. Following the 2021 introduction and subsequent spread of JEV in Australia, concern has been expressed that the presence and abundance of feral pigs may make eradication very difficult (Williams et al., 2022).

In this study, the Sinclair miniature feral phenotype pig was used as a representative model of feral pigs. Intradermal challenge with the genotype Ib JE-91 strain led to the establishment of infection, followed by the rapid onset of viremia, oronasal shedding, systemic spread, and neurotropic disease. Taken together, our findings are the first direct evidence to show that feral-domestic pig hybrids can potentially support the transmission cycle of JEV in nature.

## Materials and Methods

### Cell lines and viruses

This study used two cell lines. *Aedes albopictus* C6/36 cells were maintained in Leibovitz's L-15 medium supplemented with 10% (v/v) fetal bovine serum (FBS), 10% (v/v) tryptose phosphate broth (TPB), penicillin, streptomycin, and L-glutamine at 28°C and used for the propagation of virus stocks for the challenge experiment. African green monkey Vero76 cells were maintained in Leibovitz's L-15 medium supplemented with 10% (v/v) FBS, 10% (v/v) TPB, penicillin, streptomycin, and L-glutamine at 37°C and used to quantify the infectivity of virus stocks and tissue samples and the neutralizing activity of serum samples collected from challenged animals. JEV strain JE-91 (GenBank access number: GQ415355) was used for the challenge experiment in this study.

Strain JE-91 is representative of genotype Ib, originally isolated from mosquitoes collected in Korea in 1991 (Huang et al., 2016; Schuh et al., 2010). The virus was passaged once in African green monkey Vero cells and once in *A. albopictus* C6/36 cells before the experiments.

### Animals

Since hunting, transporting, and possessing feral pigs are illegal in Kansas (Bevins et al., 2014; Kansas, 2020), we used the Sinclair miniature feral phenotype pig as a representative model of feral pigs (Sinclair Bio Resources, Auxvasse, MO; https://sinclairbioresources.com/miniature-swine-production/sinclair-miniature-swine/). These miniature pigs are an established colony developed by the Hormel Institute at the University of Minnesota by crossbreeding four feral pig strains found in the United States (*i.e.,* Guinea hog from Alabama, wild boar from Catalina Island, Piney wood pig from Louisiana, and dwarf Ras-n-Lansa pig from Guam in the Mariana Islands) with a domestic Yorkshire boar (McAnulty, 2012; Tumbleson and Schook, 1997). It is the first strain of miniature pig developed and made available to scientists for research purposes (Bouchard et al., 1996).

Sinclair miniature feral phenotype pigs have been applied as an animal model for translational medical research in multiple field areas, such as oncology (Misfeldt and Grimm, 1994), toxicology (Brown and Hutcheson, 1973), development (Ryan et al., 2018), and metabolic disease (Stricker-Krongrad et al., 2016), as well as miniature models for diseases of conventional domestic pigs (Blagburn et al., 1991; Turnquist et al., 1993) due to their smaller size, ease in handling, and thus, lower cost associated with husbandry.

### Animal study design and sample collection

Experimental procedures and animal use were approved by the Kansas State University Institutional Biosafety and the Institutional Animal Care and Use Committees and conducted in biosafety level 3 laboratories at the Biosecurity Research Institute. Fourteen, 3-week-old Sinclair miniature feral phenotype pigs were randomly allocated into two experimental groups to be intradermally inoculated at the base of the left ear with the following: 100 μL of sterile saline (mock group, *n* = 4) or 100 μL of 10^7^ tissue culture infectious dose 50% endpoint assay (TCID_50_) of JEV JE-91 (JEV group, *n* = 10). This dosage has been proven to establish JEV infection in domestic pigs, which allows comparisons to be made (Park et al., 2021; Ricklin et al., 2016; Yamada et al., 2004). The two groups were housed in separate pens for the duration of study.

All animals were monitored daily for clinical signs, including fever (≥40°C), depression, diarrhea, weight loss, gait abnormalities, and neurological signs. Blood samples were collected daily until 7 days postinfection (dpi) and then weekly until 28 dpi to detect viremia. To characterize the viral nasal shedding, nasal swabs were obtained daily from 0 to 28 dpi from alternating nares using sterile nylon swabs and stored in 1 mL of L-15 media. Samples were processed as previously described and store in −80°C for subsequent analysis (Park et al., 2018).

To characterize the viral dissemination of JEV at the acute and convalescent stages of infection, two groups of seven pigs (five infected and two control pigs) were humanely euthanized at 3 and 28 dpi, respectively. At necropsy, ∼5 mm^3^ blocks of the following tissues were collected to be homogenized in 1 mL of L-15 media before analysis as previously described (Park et al., 2018): brain (including olfactory bulb, olfactory peduncle, piriform cortex, midbrain, pons, medulla oblongata, cerebellum, thalamus, frontal lobe, parietal lobe, temporal lobe, occipital lobe, and caudate nucleus), spinal cord (lumbosacral region), sciatic nerve, facial nerve, olfactory neuroepithelium, nasal turbinates or epithelium, thymus, tonsil, spleen, and lymph nodes (medial retropharyngeal, submandibular, mesenteric, and/or medial iliac).

### Quantification of infectious JEV and detection of the JEV genome

A standard plaque assay using Vero76 cells was performed to detect infectious virus in the serum, nasal swab, and homogenized tissue samples, as previously described (Baer and Kehn-Hall, 2014; Nuckols et al., 2015). The plaques were counted and titers were expressed in plaque forming units (PFUs)/mL or PFU/g.

Quantification of JEV genome was determined using reverse transcription-quantitative polymerase chain reaction (RT-qPCR). Genome equivalents of JEV in serum, nasal swabs, and homogenized tissues were determined using a previously published TaqMan one-step RT-qPCR assay targeting the genomic fragment encoding the nonstructural protein 5 (Pyke et al., 2004). Viral RNA was first extracted using the QIAamp viral RNA extraction kit (Qiagen) or TRIzol LS (Invitrogen) following the manufacturer's instructions. For each reaction, a standard curve was generated by 10-fold serial dilution of RNA extract derived from a JEV stock of known titer at 8.52 log_10_TCID_50_/mL. Results were reported as the genome equivalent to log_10_TCID_50_/mL (geq-TCID_50_/mL) or log_10_TCID_50_/g (geq-TCID_50_/g). Samples were considered positive when the Ct value was lower than 34.

### Plaque reduction neutralization test

To determine neutralizing antibody titers, plaque reduction neutralizing tests (PRNT) were performed as previously described (Park et al., 2021; Roehrig et al., 2008). Briefly, serum samples were first heat inactivated at 56°C for 30 min and serially diluted twofold starting from 1:10 to 1:640. Serum samples were then incubated for 1 h at 37°C with ∼75 PFUs of JEV JE-91 strain before adding onto monolayers of Vero76 cells. After 5 days of incubation, the neutralizing antibody titers were calculated based on a 50% or greater reduction in plaque counts (PRNT_50_).

### Statistical analysis

The R software (versions 3.4.1 to 4.1.0; The R Foundation) was used for data graphical display. Data collected in this study were compared with previously published data collected from domestic pigs challenged at the same dosage via the intradermal route (Park et al., 2021). All statistical tests were performed on raw data using the SPSS Statistics software (IBM) unless stated.

The Shapiro–Wilk test was used to test the normality of raw and log-transformed data. Viral loads in tissue samples collected from the experimental groups were evaluated by nonparametric Kruskal–Wallis tests with *post hoc* Dunn's multiple pairwise comparison test adjusted with Bonferroni correction (Dunn–Bonferroni test) and *post hoc* Mann–Whitney U tests. Owning to the violation of normality assumption and considering time as a factor, nonparametric Kruskal–Wallis tests and *post hoc* Dunn–Bonferroni tests were performed to compare temperature, viremia levels, and nasal shedding levels between the groups when appropriate. Mann–Whitney U tests were used to compare antibody titers and onset of ataxia between two virus-challenged groups when applicable. For the differences in the duration of nasal shedding between virus-challenged groups, Student's *t*-test was used for such an evaluation. Fisher's exact tests were used to analyze the difference in fever, nasal shedding, and ataxia incidence between the virus-challenged groups when appropriate.

## Results

### Intradermal challenge with JEV led to the onset of febrile illness and signs of neurotropic disease in Sinclair miniature feral phenotype pigs

**FIG. 1.**
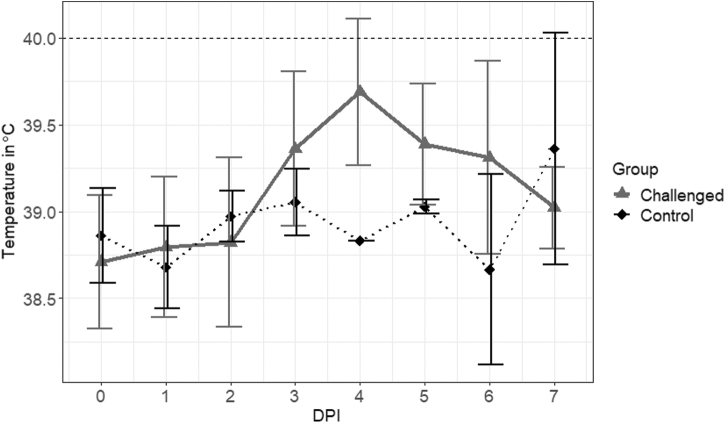
Temperature profiles of Sinclair miniature feral phenotype pigs after JEV challenge. Body temperatures were recorded from the noninfected and infected pigs. DPI, day postinfection; JEV, Japanese encephalitis virus.

Overall, the intradermal challenge of seven pigs (five infected and two control) with JE-91 strain led to the onset of transient clinical disease in the Sinclair miniature feral phenotype pigs, which resembles the acute phase (3 dpi) of JEV infection reported in domestic pigs (Park et al., 2018). With the exception of one animal that appeared depressed at 4–5 dpi, no symptoms of diseases were observed. Elevated body temperatures (≥40°C) of only 1 to 2 days of duration were recorded in 60% (3/5) of the infected pigs in the JEV-challenged group. As presented in Fig. 1, the average temperature peak in the infected pigs (39.69 ± 0.42°C) occurred at 4 dpi, in which 40% (2/5) of the pigs had elevated body temperatures of >40°C. The range of the average temperatures from day 0 to day 7 postchallenge was 38.66°C to 39.36°C for the control animals.

The convalescent phase (28 dpi) of JEV infection in the Sinclair miniature feral phenotype pigs is manifested with the development of neurotropic disease as mild-to-moderate hind limb ataxia and was observed in most infected animals assigned to study the recovery from acute symptoms. Four out of the 5 (80%) infected animals developed hind limb ataxia around 11 to 15 dpi and recovered to normal ambulation in 1 to 8 days. All infected animals survived the challenge to the end of the study and developed a mean geometric PRNT_50_ titer of 121.26.

### Onset of viremia suggests that Sinclair miniature feral phenotype pigs can facilitate the transmission of JEV by mosquitoes

**FIG. 2.**
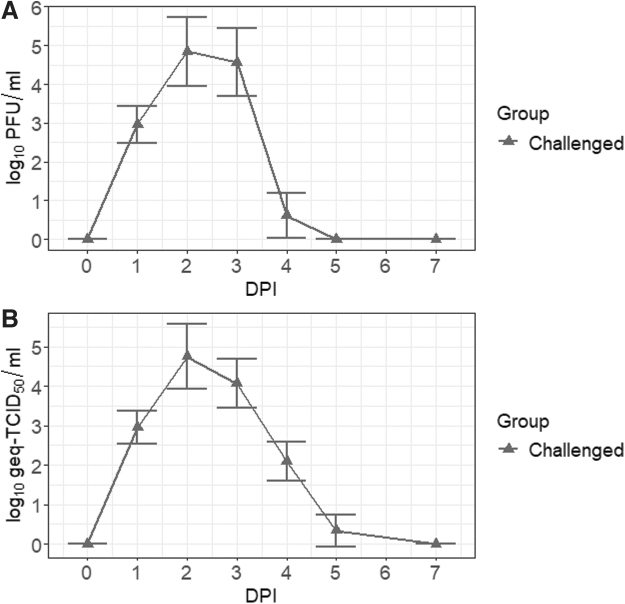
Infectivity and viral load of serum samples collected after the intradermal challenge with JEV. Infectious titers **(A)** and RNA loads **(B)** were detected in the serum by plaque assay and RT-qPCR. Geq-TCID_50_, genome equivalent-50% tissue culture infectious dose; PFU, plaque forming units; RT-qPCR, reverse transcription-quantitative polymerase chain reaction.

All infected animals developed detectable viremia, suggesting that feral pigs may serve as a source of infectious virus and play a role in the transmission of JEV. The kinetics of viremia depicted by plaque assay and RT-qPCR were consistent with each other. As shown in Fig. 2, viremia was detected at day 1 postinfection and lasted 4 to 5 days. The peak infectious titer and viral load in serum were both observed at 2 dpi. The mean infectivity and serum viral load were 7.0 × 10^4^ ± 1.1 × 10^5^ PFU/mL and 5.6 × 10^4^ ± 9.7 × 10^4^ geq-TCID_50_/mL, respectively.

### Nasal shedding of JEV during the acute phase of infection

**FIG. 3.**
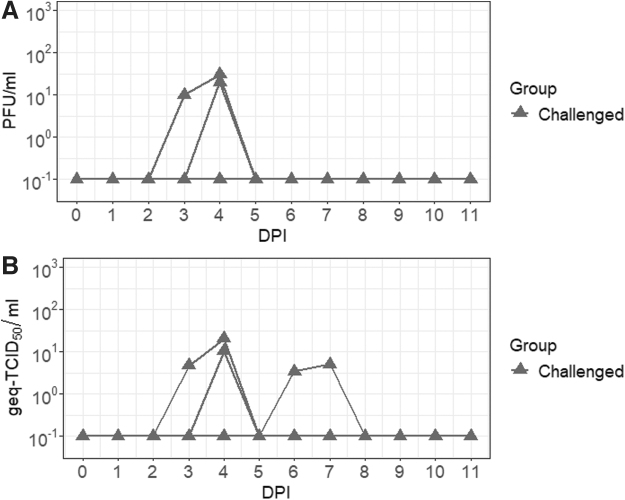
Nasal shedding of JEV in the Sinclair miniature feral phenotype pigs. Viral infectious titers **(A)** and RNA loads **(B)** were detected in the nasal swabs from the infected pigs.

Infectious virus and RNA genome were detected in the nasal secretions of the Sinclair miniature feral phenotype pigs during the acute phase of infection as depicted in Fig. 3. Infectious virus was detected in 40% (2/5) of the animals with an average infectivity of 1.8 × 10^1^ ± 1.0 × 10^1^ PFU/mL between 3 and 5 dpi. Data analyzed by RT-qPCR provided a higher sensitivity of detection than plaque assay. Viral genome first became detectable as early as 2 dpi and persisted up to 8 dpi. The highest viral load was 6.1 ± 8.2 geq-TCID_50_/mL at 4 dpi. No positive JEV detection was made after 7 dpi.

### Viral burdens in different tissues at the acute phase of infection

**FIG. 4.**
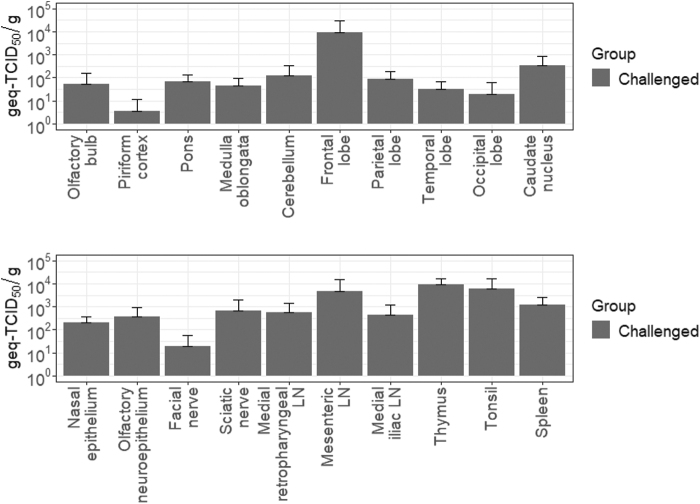
Tissue dissemination pattern of JEV in Sinclair miniature feral phenotype pigs after challenge. Viral RNA loads were detected in several different tissues collected at day 3 postinfection from the infected pigs.

Evidence of systemic dissemination of JEV at the acute phase of infection was demonstrated based on the detection of infectious virus and JEV RNA in different tissues at 3 dpi. Most notably, infectious virus and viral RNA were present in central nervous system (CNS) tissues, proving that JEV exhibits the neuroinvasive phenotype in feral pigs. Infectious titers of homogenized nervous and lymphoid tissues ranged from 2.6 × 10^1^ ± 3.2 × 10^1^ PFU/g (medial iliac lymph node) to 5.4 × 10^3^ ± 1.0 × 10^4^ PFU/g (mesenteric lymph node). Analysis by RT-qPCR validated the results from the plaque assay, as shown in Fig. 4. There were no demonstrable differences in the infectious titers or viral loads between different lymphoid and CNS tissues.

### Lymphoid tissues support the persistent infection of JEV

**FIG. 5.**
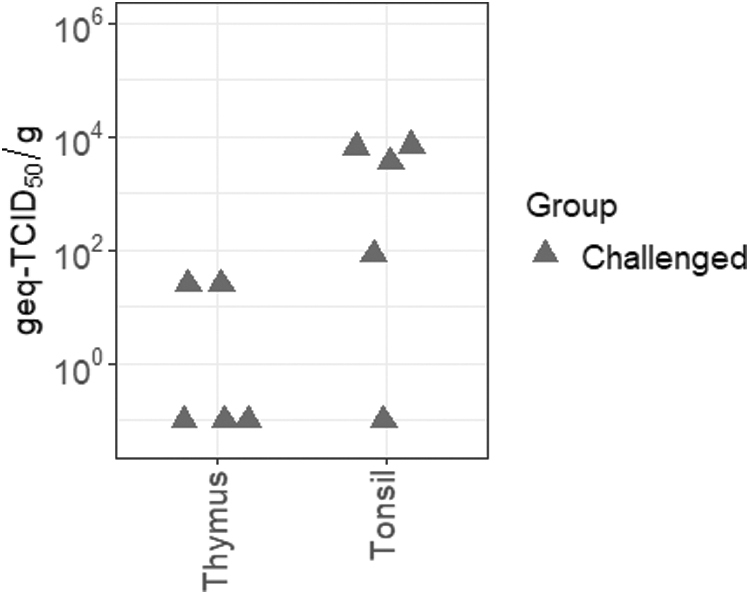
Persistent infection of lymphoid tissues collected from JEV-infected Sinclair miniature feral phenotype pigs. Viral RNA loads were detected in lymphoid tissues collected at day 28 postinfection from the infected pigs.

Based on the detection of JEV RNA in tissues collected at 28 dpi by RT-qPCR, persistent infection of JEV was demonstrated in the Sinclair miniature feral phenotype pigs, suggesting that they can also be used as an additional model of JEV persistence. Persistent infection of the tonsils and thymus was demonstrated in the JEV-infected pigs. As presented in Fig. 5, viral RNA loads were detected in both lymphoid structures of the infected pigs. Mean viral loads of the thymus was 9.9 × 10^0^ ± 1.2 × 10^1^ geq-TCID_50_/g. The average viral loads of the tonsil were slightly higher than the thymus, reaching 3.4 × 10^3^ ± 3.0 × 10^3^ geq-TCID_50_/g.

## Discussion

To date, domestic pigs have been considered important reservoirs for various human pathogens, including JEV and hepatitis E virus. While domestic and wild or feral pigs are evolutionarily related, few studies have investigated whether or not genetic differences between them could be translated to different susceptibilities and consequently the role in enzootic transmission (Conyers et al., 2012; Costa et al., 2012; Lowden et al., 2002). Such knowledge can be crucial for the broader picture of JEV epidemiology, as several of the endemic countries do not have large-scale pig farming, which are the most common source of epizootic spillover. There is little demonstrable difference between the Sinclair miniature feral phenotype pigs and domestic pigs in clinical signs, viremia, oronasal shedding, and neuroinvasive phenotype during the acute phase of infection, following the intradermal challenge with JEV (Park et al., 2018; Ricklin et al., 2016; Yamada et al., 2004).

Because the virus was delivered through the intradermal route, the development of viremia clearly indicates that JEV can replicate and disseminate in the Sinclair miniature feral phenotype pigs. The peak viral burden in the serum of feral pigs at 2 dpi resembled the viremic kinetics observed in domestic pigs (Park et al., 2021). Therefore, crossbreeding with feral pigs has no demonstrable impact on the susceptibility of domestic pigs to JEV. Hybrids between domestic pigs and feral pigs that exist in large numbers in nature can also serve as an amplifying host for the transmission of JEV by mosquitoes.

Based on the high seroprevalence reported in feral pigs captured in the endemic areas (Hamano et al., 2007; Nidaira et al., 2014; Nidaira et al., 2007; Ohno et al., 2009; Yang et al., 2012), we speculate that the transmission of JEV to feral pigs by mosquitoes is likely to take place frequently in nature. This indicates that transmission of JEV by *S. scrofa* may be more prevalent than previously thought, as feral pigs may also serve as an amplifying host for JEV in regions that have few domestic pigs (Kumar et al., 2018; Kuwata et al., 2020).

In this study, we used 3-week-old crossbred Sinclair miniature feral phenotype pigs as a model to demonstrate the susceptibility of feral pigs to JEV. The observations may not be directly extrapolated to the disease pathogenesis of JEV in adult wild or feral pigs in nature as the susceptibility of feral pigs to swine pathogens often is affected by age (Kaden et al., 2004). For example, young wild boars frequently developed longer viremia of classical swine fever virus, whereas the viremia was often short and transient in adult wild boars (Kaden et al., 2004). Nevertheless, the susceptibility of young feral pigs to JEV remains important and relevant as there are continuously stable populations of piglets due to the high turnover rate of feral pigs in nature similar to domestic pigs in swine or pork production.

Among the minor differences in infection outcomes observed in our study, the duration of nasal shedding of JEV RNA was found to be significantly shorter in the Sinclair miniature feral phenotype pigs compared with domestic pigs (Park et al., 2021). However, this finding may be due to differences in sampling techniques. At the same ages (*i.e.,* 3 weeks old before the start of the study), the Sinclair miniature feral phenotype pigs were much smaller in size than the domestic pigs used in the Park et al.'s (2021) study. Due to the size difference, pediatric-sized nylon swabs were used for the duration of this study instead of the regular-sized cotton swabs that were used for the domestic pigs in our previous study (Park et al., 2021) to fit the smaller nostrils of the feral pigs for the nasal swab collection.

This could result in less virus recovered from the swabs, leading to a lower yield of virus and viral RNA. It is also unknown if vector-free transmission also applies to feral pigs. JEV RNA has only been detected in the serum (Nidaira et al., 2008) and tonsils (Tan et al., 2012) of wild pigs from JE-endemic regions. At this time, no infectious JEV has been isolated from wild or feral pigs. The ability of JEV-infected feral pigs to transmit the virus through direct nose-to-nose contact with other naive feral or domestic pigs remains unknown but can potentially have significant implications as contact between feral and domestic pigs in outdoor pens is a common occurrence (Miller et al., 2017; Wyckoff et al., 2009). This potential route of transmission should be investigated in the future.

Another difference found between the current study and our previous study with domestic pigs was a lower viral burden in two CNS tissues sampled from the Sinclair miniature feral phenotype pigs at day 3 postinfection, including the piriform cortex and occipital lobe (Park et al., 2021). However, the viral burden in the rest of the CNS and lymphoid tissues was comparable between the two groups of animals. Overall, the infection and clinical disease course, including viremia kinetics, tissue tropism, and viral persistence, was similar and indistinguishable when compared between both domestic and the Sinclair miniature feral phenotype pigs, suggesting that different biotypes of *S. scrofa* are uniformly susceptible to JEV.

To our best knowledge, this project was the first to prove that hybrids between domestic and feral pigs are susceptible to JEV infection. The Sinclair miniature feral phenotype pigs provide a biologically relevant model for JEV pathogenesis in feral pigs, which has been proven challenging to investigate. Importantly, our findings suggest that at least two subspecies of *S. scrofa* are susceptible to JEV and the detailed understanding of JEV pathogenesis in different subspecies of *Sus scorfa* will contribute to a more complete picture of JEV epidemiology and ecology.
